# Risk factors for low birth weight in Nigeria: evidence from the 2013 Nigeria Demographic and Health Survey

**DOI:** 10.3402/gha.v9.28822

**Published:** 2016-01-19

**Authors:** Maznah Dahlui, Nazar Azahar, Oche Mansur Oche, Norlaili Abdul Aziz

**Affiliations:** 1 Centre of Population Health, Department of Social and Preventive Medicine, Faculty of Medicine, University of Malaya, Kuala Lumpur, Malaysia; 2 Department of Medical Laboratory Technology, Faculty of Health Sciences, Universiti Teknologi MARA, Pulau Pinang Campus, Malaysia; 3 Department of Community Health, Usmanu Danfodiyo University, Sokoto, Nigeria

**Keywords:** low birth weight, Nigeria, risk factors, maternal and child health

## Abstract

**Background:**

Low birth weight (LBW) continues to be the primary cause of infant morbidity and mortality.

**Objective:**

This study was undertaken to identify the predictors of LBW in Nigeria.

**Design:**

The data for this study was extracted from the 2013 Nigeria Demographic and Health Survey conducted by the National Population Commission. Several questionnaires were used in the survey, some covering questions on pregnancy characteristics. The inclusion criteria include mothers who gave birth to a child 5 years before the interview and aged 15–49 years who were either permanent residents or visitors present in the household on the night before the survey conducted. The birth weight of the infants was recorded from written records from the hospital cards or the mothers’ recall.

**Results:**

The prevalence of LBW in this study was 7.3%. Multiple logistic regression analysis showed an adjusted significant odds ratio for mothers from North West region (aOR 10.67; 95% CI [5.83–19.5]), twin pregnancy (aOR 5.11; 95% CI [3.11–8.39]), primiparous mother (aOR 2.08; 95% CI [1.15–3.77]), maternal weight of less than 70 kg (aOR 1.92; 95% CI [1.32–2.78]), and manual paternal employment (aOR 1.91; 95% CI [1.08–3.37]).

**Conclusions:**

The risk factors for LBW identified in this study are modifiable. In order to reduce this menace in Nigeria, holistic approaches such as health education, maternal nutrition, improvement in socio-economic indices, and increasing the quality and quantity of the antenatal care services are of paramount importance.

## Introduction

Birth weight is one of the significant predictors of child mental development, future physical growth, and survival. It is one of the important risk factors for child morbidity and mortality (1–4). According to the World Health Organization (WHO), low birth weight (LBW) is defined as an infant birth weight of less than 2,500 g (5). This group of children is considered to have higher risk of neonatal, post-neonatal death, and morbidity (6). Infant with LBW is associated with early and late morbid conditions such as impaired cognitive function (7), psychological disorders (8), and coronary heart disease (9). The factors for LBW are yet to be completely understood even though abundant research has been conducted to ascertain the underlying factors. Although LBW is considered as a multifactorial disease, most of the risk factors are preventable before pregnancy.

There is significant difference in the incidence of LBW between developed and developing countries and between various regions in a country. In developed countries, the occurrence is 7%, while in developing countries it is 15% (10). Globally, recent estimates suggest that there were 18 million of LBW babies born every year (11). In sub-Saharan Africa, the prevalence of LBW varies according to the regions. The prevalence of LBW in Ethiopia was 28.3% (12) while there were 199 LBW infants per 1,000 live births in Zimbabwe (13). In Nigeria, LBW affects about 5–6 million children every year (14). The incidence was 12.1% in Jos (15), 11.4% in Ogun (16), and 16.9% in Maiduguri (17). A number of factors need to be investigated in order to lessen the prevalence of LBW in Nigeria.

There are numerous maternal and fetal factors contributing to the LBW incident (18). LBW is strongly associated with maternal factors such as younger and older age, low socio-economic status, residence in the rural area, and illiteracy (11, 19–22). Mothers aged under 17 and over 35 years are at risk of delivering LBW babies (23). Mothers in deprived socio-economic conditions frequently have LBW infants (12). There is ample evidence to show that maternal factors and risk behaviors during antenatal period play significant roles in the birth weight of babies (24, 25). Pregnant mothers with unhealthy lifestyles that include activities such as smoking were found to be at high risk of delivering LBW babies (26). A previous study also had showed that drugs taken during pregnancy, such as malaria prophylaxis, were associated with the incidence of LBW (17). Other risk factors linked with LBW include maternal height (27), body mass index (BMI) (28), weight (29), parity (26), birth interval (30), multiple gestation, the experience of any physical violence (12), and the lack of skilled antenatal care (31). Paternal factors such as level of education (11), age (30), and employment (31) were also significantly linked to the incidence of LBW.

Antenatal care (ANC) visits are important for maternal and fetus health. ANC refers to pregnancy-related healthcare services provided by skilled health personnel during pregnancy that monitor the well-being of both the mother and the unborn child. It is essential to the purposes of obtaining the best possible outcome and preventing any complications (32). The frequency of ANC visits and parity are significantly associated with birth outcomes such as birth weight (17, 33). Pregnant mothers who attended less than four ANC visits double their risk of delivering LBW babies compared to those visiting four or more times (34, 35). Also, studies found that the prevalence of LBW was high, up to 57% (36) and 61.8% (37), among mothers who did not receive any ANC. Due to the irregularity of ANC visits, pregnant mothers do not comply with the advice or medications recommended by healthcare providers and subsequently will increase the incidence of LBW (38). The quality of each ANC visit also should be emphasized in order to have an effective coverage of care.

LBW is one of the most important public health concerns worldwide and is still the leading cause of prenatal and neonatal deaths. Despite intense research conducted on LBW globally, the factors affecting LBW in Nigeria have not been adequately investigated. Identifying the predictors of LBW and addressing the best prevention strategies will help to avert early the childhood morbidity and mortality resulting from LBW. Therefore, this study was aimed at 1) determining the prevalence of LBW infants in Nigeria, 2) describing the socio-demographic and pregnancy characteristics of mothers who gave birth to LBW babies, and 3) exploring the predictors of LBW in Nigeria using the 2013 Nigeria Demographic and Health Survey (2013 NDHS).

## Methods

This manuscript had been developed by utilizing the 2013 NDHS data obtained from the Ministry of Health, Nigeria. NDHS was a cross-sectional survey with a stratified three-stage cluster design. It is the fifth in the series of national surveys implemented by the National Population Commission (NPC). This nationally representative survey covered all 36 states and the Federal Capital Territory in Nigeria. This survey was intended to provide the latest estimates on population and health in Nigeria. The list of enumerated areas prepared for the 2006 Population Census of the Federal Republic of Nigeria was used as to frame the samples. The selection of samples was based on stratified three-stage cluster design which consists of 372 clusters in urban areas and 532 clusters in rural areas, giving a total of 904 clusters. A total of 40,320 households were selected. The inclusion criteria for this study were mothers, aged 15–49 years, who gave birth to a child 5 years before the interview and were either permanent residents or current visitors in the household on the night before the survey conducted. Data collection was carried out between February and May 2013.

A structured questionnaire was used for interviewing the mothers. The questionnaire captured information about the pregnancies such as maternal age, birth interval, parity, time of registration, and frequency of ANC visits from mothers. Socio-demographic details of the mothers such as highest educational attainment, wealth index, localities, and literacy levels were also obtained. Infant characteristics such as sex and mode of delivery were recorded. The questionnaire was translated into different Nigerian languages: Hausa, Igbo, and Yoruba. All questionnaires were pretested among 120 households in November 2012. The questionnaires were modified according to the country's requirement with the advice from health experts.

Prior to the commencement of the study, a complete listing of households was obtained and a mapping exercise was conducted for each cluster from December 2012 until January 2013. Training on how to use the Global Positioning System (GPS) receivers to locate the coordinates of sample households was conducted for the enumerators. All participants were briefed on the objectives, procedures, expected outcomes, benefits, and risk associated with this study. An informed consent was obtained from the mothers prior to the interview. Ethical clearance to conduct NDHS was approved by the National Health Research Ethics Committee of Nigeria, Federal Ministry of Health, Abuja, Nigeria. NDHS data are public access data and were made available to us upon request.

Birth weights of the infants were recorded based on the written records of the hospital cards or mothers’ recall. Birth weights less than 2,500 g were classified as LBW (5). Independent variables studied in this survey were selected based on previous literature reviews on LBW (32, 39–41). Age was recorded as continuous variable and then recoded into categories. Education was recorded as 1) no education, 2) primary, and 3) secondary or higher education. Occupation was categorized as 1) unemployed and 2) employed. Household wealth index was divided into quintiles according to the wealth score, 1) poorest, 2) poor, 3) average, 4) rich, and 5) richest. However, due to similarities between some quintiles, the wealth index has been re-categorized into 1) poor, 2) middle, and 3) rich (42). The heights and weights of the mothers were also measured. Measurements were done using lightweight SECA scales (with digital screens). The measuring boards used in this study were designed by Shorr Productions for use in the survey settings. Maternal height less than 1.45 m (32) and weight 70 kg (40) were chosen as cut off for this study.

Birth order was recorded as continuous variable and then recoded as 1) first and 2) second or more. Information on the number of children ever born or parity was obtained. The period of time between two successive live births or birth interval was recorded. Place of birth was dichotomized into 1) delivery at health facility and 2) delivery at home. The frequency of ANC visits was grouped into 1) less than 4 visit and 2) ≥4 visits. The proportion of pregnant mothers who received four or more ANC visits has been used as a benchmark for adequate ANC (43). In addition, pregnant mothers were asked about the timing of their first ANC visit and were classified as 1) early ANC registration (within the first trimester) and 2) late ANC registration (after the first trimester). The percentage of pregnant mothers who took malaria prophylaxis and intestinal parasite drugs during pregnancy was recorded. According to Kayode et al. (39), pregnancies were classified as 1) wanted then, 2) wanted no more, and 3) wanted later. Mothers also were asked whether they had ever experienced physical violence during pregnancy.

The Statistical Package for the Social Sciences (SPSS) version 20 with a complex samples procedure was used for the analysis. Frequency and percentages with 95% confidence interval were used to describe the characteristics of the LBW babies. The chi-square analysis procedure in the complex samples add-on module in SPSS was used to test the associations between socio-demographic and pregnancy characteristics with birth weight of the babies. *P*-value < 0.05 indicates significant association. The multivariate logistic regression procedure was used to determine the predictors of LBW babies in Nigeria. Significant predictors were identified based on 95% CI. All variables were tested in a simple model using logistic regression to obtain the unadjusted logistic regression. All factors for LBW were included in the model. The variables were entered using manual stepwise method. All significant variables reaching *p*<0.05 were retained in the model.

## Results

There were 5,189 babies who were weighed after delivery during the 5 years preceding the survey. The proportion of LBW in this study was 7.3%. Among LBW babies, 39.5% were from written records from the hospital cards.

The results for the socio-demographic characteristics of mothers who delivered LBW babies are presented in Table 1. LBW is significantly higher among children whose mothers were aged 15–24 years (11.5%) and mothers without formal education (14.9%). About 10% of the unemployed and non-married mothers gave birth to LBW babies. The birth weights of infants varied by geopolitical zone. The North West region had the highest proportion of LBW, a significant 27.2% of the population. Among different ethnic groups, Hausa mothers had a significantly higher proportion of LBW compared to other ethnic groups. The proportion of LBW was significantly higher among mothers with poor wealth indices (16.7%) and was also more common in the rural areas (9.7%). There was also significant association between paternal educational level and LBW.

**Table 1:** Association between birth weight and socio-demographic characteristics

Factors	Total baby weighed *n* (%)	Low birth weight% (95% CI)	p*
Maternal age (years)			
15–24	761 (14.5)	11.5 (8.9, 14.8)	0.014^b^
25–34	3,067 (60.2)	7.8 (6.7, 9.0)	
35–49	1,361 (25.3)	7.2 (5.6, 9.1)	
Maternal education			
No education	290 (5.6)	14.9 (10.8, 20.2)	0.002^b^
Primary	799 (14.9)	8.0 (5.9, 10.8)	
Secondary or higher	4,100 (79.5)	7.7 (6.7, 8.8)	
Maternal employment			
Unemployed	1,112 (21.2)	10.0 (7.9, 12.5)	0.056
Employed	4,077 (78.8)	7.7 (6.7, 8.8)	
Paternal age (years)			
16–35	1,825 (37.7)	8.2 (6.8, 10.0)	0.852
36–55	2,900 (59.3)	8.0 (6.8, 9.3)	
More than 55	170 (3.1)	8.0 (7.1, 9.1)	
Paternal education			
No education	193 (3.7)	14.4 (9.3, 21.5)	0.015^b^
Primary	858 (16.7)	6.8 (5.1, 9.1)	
Secondary or higher	3,988 (79.6)	8.1 (7.0, 9.2)	
Paternal employment			
Others	1,814 (35.5)	7.1 (5.8, 8.7)	0.484
Manual	1,668 (34.5)	8.7 (7.1, 10.7)	
Agricultural	524 (8.3)	8.6 (6.0, 12.1)	
Sales	1,034 (21.7)	8.5 (6.6, 10.8)	
Marital status			
Married	4,698 (90.8)	7.9 (7.0, 9.0)	0.112
Non-married	491 (9.2)	10.5 (7.6, 14.4)	
Wealth index			
Poor	290 (5.1)	16.7 (12.0, 23.0)	<0.001^c^
Middle	2,190 (39.7)	8.8 (7.4, 10.5)	
Rich	2,709 (55.2)	6.9 (5.8, 8.2)	
Religion^a^			
Catholic and other Christian	3,872 (75.1)	8.3 (7.2, 9.5)	0.673
Islam	1,288 (24.9)	7.9 (6.4, 9.7)	
Ethnicity			
Others	1,796 (32.7)	12.5 (10.6, 14.6)	<0.001^c^
Igbo	1,613 (33.3)	6.0 (4.7, 7.7)	
Hausa	307 (5.5)	15.4 (11.2, 21.0)	
Yoruba	1,473 (28.5)	4.3 (3.2, 5.8)	
Residence type			
Urban	3,616 (73.1)	7.6 (6.6, 8.8)	0.048^b^
Rural	1,573 (26.9)	9.7 (8.0, 11.7)	
Geopolitical zone			
North Central	771 (10.2)	7.5 (5.5, 10.1)	<0.001^c^
North East	374 (5.8)	13.0 (9.6, 17.4)	
North West	308 (9.4)	27.2 (21.8, 33.3)	
South East	1,299 (25.2)	4.7 (3.5, 6.3)	
South South	850 (15.3)	11.5 (9.1, 14.4)	
South West	1,587 (34.1)	3.4 (2.5, 4.6)	

* The *p*-value is based on chi-square test.

a Percentages are calculated based on less than 5,189 infants

b *p*<0.05

c *p*<0.001.

Results for the pregnancy characteristics of mothers who gave birth to LBW babies are shown in Table 2. The number of previous pregnancies, maternal weight and height, frequency of ANC visit, maternal BMI, and the experience of physical violence during pregnancy were significantly associated with LBW. Among the LBW, there was a greater proportion of multiparous and maternal weight less than 70 kg. More than 80% of the mothers with LBW were multiparous and had weight less than 70 kg. More than half of the LBW, representing 61.7%, were babies born to mothers with normal BMI. Single pregnancy, maternal height ≥1.45 m, ≥4 ANC visits and no experience of any physical violence during pregnancy were associated with a significantly higher proportion of LBW.

**Table 2:** Association between birth weight and pregnancy characteristics of mothers

Factors	Total baby weighed *n* (%)	Low birth weight% (95% CI)	p*
Birth order			
First child	1,427 (27.7)	28.0 (22.9, 33.8)	0.889
Second or more child	3,762 (72.3)	72.0 (66.2, 77.1)	
Birth interval (months)^a^			
< 18	278 (7.5)	7.9 (4.7, 12.8)	0.536
18–36	2,005 (53.1)	49.2 (42.0, 56.5)	
> 36	1,458 (39.3)	42.9 (36.0, 50.2)	
Place of birth^a^			
Home delivery	369 (7.3)	8.9 (6.3, 12.5)	0.242
Health facility delivery	4,807 (92.7)	91.1 (87.5, 93.7)	
Parity			
Primiparous	877 (17.0)	19.5 (15.1, 24.9)	0.252
Multiparous	4,312 (83.0)	80.5 (75.1, 84.9)	
Mode of birth^a^			
Vaginal birth	4,772 (92.8)	91.8 (87.9, 94.5)	0.486
Caesarean birth	369 (7.2)	8.2 (5.5, 12.1)	
Number of pregnancy			
Single	4,986 (96.0)	89.6 (85.8, 92.4)	<0.001^c^
Twin	203 (4.0)	10.4 (7.6, 14.2)	
Sex of infant			
Male	2,629 (49.9)	47.6 (41.6, 53.6)	0.436
Female	2,560 (50.1)	52.4 (46.4, 58.4)	
Maternal height (m)^a^			
Less than 1.45 m	26 (0.5)	1.5 (0.5, 4.3)	0.012^b^
1.45 m and above	5,126 (99.5)	98.5 (95.7, 99.5)	
Maternal weight (kg)^a^			<0.001^c^
Less than 70 kg	3,498 (67.3)	80.2 (75.0, 84.6)	
70 kg and above	1,664 (32.7)	19.8 (15.4, 25.0)	
Maternal BMI^a^			
Underweight	171 (3.2)	3.2 (1.7, 6.1)	<0.001^c^
Normal weight	2,542 (49.5)	61.7 (55.7, 67.3)	
Overweight	1,572 (30.8)	24.0 (19.4, 29.4)	
Obese	852 (16.5)	11.1 (7.8, 15.4)	
Maternal smoking status^a^			
Non-smoker	5,140 (99.6)	99.9 (99.5, 100.0)	0.069
Smoker	28 (0.4)	0.1 (0.0, 0.5)	
Frequency of ANC visit^a^			
Less than 4 visits	353 (6.8)	12.6 (9.2, 16.9)	<0.001^c^
≥ 4 visits	4,579 (93.2)	87.4 (83.1, 90.8)	
Timing of ANC visit^a^			
Early ANC registration	1,661 (33.1)	28.6 (23.4, 34.5)	0.109
Late ANC registration	3,441 (66.9)	71.4 (65.5, 76.6)	
Index pregnancy wanted^a^			
Wanted then	4,584 (88.3)	87.4 (83.1, 90.7)	0.145
Wanted later	455 (8.8)	11.1 (8.0, 15.2)	
Wanted no more	140 (2.8)	1.5 (0.6, 3.8)	
Use of malaria prophylaxis during pregnancy^a^			
No	1,078 (29.4)	28.7 (22.6, 35.7)	0.847
Yes	2,645 (70.6)	71.3 (64.3, 77.4)	
Drugs for intestinal parasite during pregnancy^a^			
No	2,963 (79.2)	75.7 (69.2, 81.2)	0.216
Yes	797 (20.8)	24.3 (18.8, 30.8)	
Experience any type of violence during pregnancy^a^			
No	2,995 (93.2)	88.2 (81.4, 92.7)	0.015^b^
Yes	172 (6.8)	11.8 (7.3, 18.6)	

* The *p*-value is based on chi-square test

a Percentages are calculated based on less than 5,189 infants

b *p*<0.05

c *p*<0.001.

The determinants of each variable were presented in Table 3. The determinants for LBW in Nigeria were paternal employment, geopolitical zone, parity, number of pregnancies, and maternal weight. Mothers from North West region were 10.67 times more likely to deliver LBW (aOR 10.67; 95% CI [5.83–19.5]). Primiparous mothers were 2.08 times more likely to deliver a LBW baby (aOR 2.08; 95% CI [1.15–3.77]). Twin pregnancy (aOR 5.11; 95% CI [3.11–8.39]), maternal weight of less than 70 kg (aOR 1.92; 95% CI [1.32–2.78]), and manual paternal employment (aOR 1.91; 95% CI [1.08–3.37]) were more likely to be associated with LBW.

**Table 3:** Unadjusted and adjusted odds ratio (95% confidence interval [95% CI]) for factors associated with LBW

	Univariable logistic regression	Multivariable logistic regression
	
Factors	Odds ratio (95% CI)	
Paternal employment		
Agricultural	1.00	1.00
Others	0.81 (0.53, 1.26)	1.27 (0.69, 2.33)
Manual	1.02 (0.66, 1.58)	1.91 (1.08, 3.37)
Sales	0.98 (0.62, 1.57)	1.63 (0.88, 3.04)
Geopolitical zone		
South West	1.00	1.00
North Central	2.28 (1.45, 3.60)	2.45 (1.46, 4.13)
North East	4.23 (2.65, 6.75)	4.93 (2.66, 9.15)
North West	10.56 (6.87, 16.22)	10.67 (5.83, 19.50)
South East	1.39 (0.89, 2.16)	1.09 (0.55, 2.15)
South South	3.65 (2.42, 5.51)	1.95 (1.07, 3.55)
Parity		
Multiparous	1.00	1.00
Primiparous	1.21 (0.87, 1.67)	2.08 (1.15, 3.77)
Number of pregnancy		
Single	1.00	1.00
Twin	3.31 (2.22, 4.94)	5.11 (3.11, 8.39)
Maternal weight (kg)		
70 kg and above	1.00	1.00
Less than 70 kg	2.08 (1.53, 2.83)	1.92 (1.32, 2.78)

## Discussion

Information on infants’ birth weight and size at birth is essential to avert the complications resulting from LBW. In the present study, 5,189 birth weights of children were measured in the 5 years preceding the survey. This is not surprising because majority of the births did not take place in the health facility, and children are less likely to be weighed at birth in a non-institutional setting. The prevalence of LBW in this study was 7.3%, which is lower than the global prevalence in developed countries (17%) (44). The prevalence of LBW in India is higher than the current prevalence in Nigeria. Studies from two medical colleges and one civil hospital in India found that the prevalence of LBW was 26.8% (45). This is expected as the study was conducted among pregnant mothers in tertiary care, where many high-risk pregnant mothers are referred. In addition, significant predictors of LBW were identified. Paternal employment, geopolitical zone, parity, number of earlier pregnancies, and maternal weight were significant predictors of LBW in Nigeria.

This study has shown that maternal age and education, paternal education, wealth index, ethnicity, type of residence, and geopolitical zone were significantly associated with the birth weights of the infants. The association of birth weight with locality, wealth index, and maternal education observed in this study has also been reported by Jayant et al. (32). Lower wealth index, maternal education, and mothers who reside in rural areas were significantly associated with a higher percentage of infants with LBW. Many studies have shown that low levels of educational attainment were predictors of adverse birth outcomes, such as preterm birth and LBW (46–48). It is noted that higher level of education could improve the socio-economic status of the family and subsequently the odds for delivering LBW infants could be reduced. Education will guide the pregnant mothers to make decisions about their reproductive health and improve their interactions with the healthcare system (49).

The proportion of LBW children in the rural areas was higher than that of the urban areas in this study. It could be due to the fact that rural women are more susceptible to poor diet, infections during pregnancy, and inadequate ANC facilities (32). The proportion of LBW was higher among younger mothers compared to other age groups. Our findings were similar to those of the study conducted in India (29). Pregnancy at a young age is detrimental to the health of both mother and the unborn child. Among teenage mothers, the physical development of the girl is still not complete (50). Indeed, most of the younger mothers were unprepared, unaware or inexperienced (51).

With regards to pregnancy characteristics, our study found that number of earlier pregnancies, maternal height, weight and BMI, the experience any type of violence during pregnancy, and the frequency of ANC visits were significantly associated with the birth weights of the infants. The majority of the LBW infants were born to mothers who were late for their ANC registration. Inadequate ANC increases the risk of delivering LBW infants (45). Access to high-quality ANC should be highlighted since it not only enhances maternal health, but also creates opportunities for counseling and risk detection. Risk factors for LBW should be identified during ANC visits. Through this initiative, numerous opportunities exist during pregnancy to minimize the risk of LBW. This study had also identified that maternal weight was significantly associated with LBW in Nigeria. There is ample evidence to show that maternal weight is related to LBW (29, 38).

Logistic regression analysis was computed to determine the predictors of LBW in Nigeria. Among the factors tested, five risk factors were identified as significant for LBW. The risk factors were geopolitical zone, primiparous women, twin pregnancy, maternal weight less than 70 kg, and paternal employment. The most important risk factor associated with LBW in Nigeria was pregnant mothers living in the North West region. This might result from the facts that the presence of skilled attendants at births is particularly low and the number of teenage pregnancy is high in this region (52). Mothers with weight less than 70 kg were two times at risk for delivering LBW babies. It has been proven that maternal stature is one of the significant risk factors for LBW (29), underscoring the need to improve the nutritional status of women, especially during adolescence, in order to ensure that they have ideal weights. Hence, the risk factors for LBW could be reduced. This study also showed that primiparous women had two times the usual risk of delivering a LBW baby. Documented evidence has observed that being a primiparous woman is one of the risk factors for LBW (53, 54). Indeed, most of the primiparous women were young, with the subsequent increased risk of having a LBW baby. Pregnant mothers with twin pregnancies were more prone to having LBW babies. Twin pregnancy has been well-recognized as a risk factor for LBW (55), possibly because all the aspects related to fetal growth are shared between two fetuses (41).

Pregnant women exposed to tobacco products were at high risk of delivering LBW babies (45). However, in the present study, smoking status was not found to be a significant risk factor for LBW. The number of smoking pregnant mothers in this study was very low. Information on birth weight or size at birth is important for the design and implementation of programs aimed at reducing neonatal and infant mortality. Our study suggests that there are still a number of factors for LBW not studied in this survey that should be assessed in the future. Factors like poor maternal nutritional status during the antenatal period (12), history of abortion (32), pregnancy-induced hypertension (56), gestational age (38), and anemia (36) should be investigated in the future.

LBW is an indicator of infant's survival, growth, and psychosocial development (57). In the light of this sequela, the early detection of at-risk pregnancies, together with intensive ANC is crucial.

The result of the current study had provided valuable information on the significant risk factors associated with LBW infants, based on the recent national survey in Nigeria. The findings from this study will provide insight for public health professionals and policy makers to implement strategies or intervention programs to reduce the prevalence of LBW in the future. Our study has some limitations. Recall bias is possible during data collection. Birth weights of the infants were obtained from written records from the hospital cards or mothers’ recall. Moreover, a low number of LBW babies were recorded in health facilities, because most births do not take place in these facilities, which may have resulted in an underestimation of the prevalence of LBW in this study.

## Conclusions

Paternal employment, geopolitical zone, parity, number of pregnancies, and maternal weight were the significant factors for LBW in Nigeria. Even though LBW is influenced by a multiplicity of factors, the incidence of LBW could be reversed if maternal risk factors are detected earlier and appropriate prevention strategies are delivered to the high-risk group. From a public health perspective, it is an advantage that most of these factors can be modified.

Improvement in the ANC services in Nigeria is needed. Multi-faceted approaches could deliver better services to the pregnant mothers in Nigeria. Such approaches would include health education, maternal nutrition, improvement in socio-economic indices, and more and better-quality ANC services.
